# Draft genome sequences for *Neonectria magnoliae* and *Neonectria punicea,* canker pathogens of *Liriodendron tulipifera* and *Acer saccharum* in West Virginia

**DOI:** 10.1128/mra.01042-24

**Published:** 2024-11-27

**Authors:** Hannah M. Petronek, Matt T. Kasson, Amy M. Metheny, Cameron M. Stauder, Brian Lovett, Shannon C. Lynch, Jeff R. Garnas, Lindsay R. Kasson, Jason E. Stajich

**Affiliations:** 1 School of Natural Resources and the Environment, West Virginia University, Morgantown, West Virginia, USA; 2 U.S. Department of Agriculture Forest Service, Forest Health Protection, Bend, Oregon, USA; 3 U.S. Department of Agriculture Agricultural Research Service, Emerging Pests and Pathogens Research Unit, Ithaca, New York, USA; 4 Department of Plant Pathology, University of California-Davis, Davis, California, USA; 5 Department of Natural Resources and the Environment, University of New Hampshire, Durham, New Hampshire, USA; 6 School of Medicine, West Virginia University, Morgantown, West Virginia, USA; 7 Department of Microbiology and Plant Pathology, University of California-Riverside, Riverside, California, USA; Rochester Institute of Technology, Rochester, New York, USA

**Keywords:** fungi, Nectriaceae, canker pathogens, Hypocreales, genomics

## Abstract

The fungal genus *Neonectria* contains many phytopathogenic species currently impacting forests and fruit trees worldwide. Despite their importance, a majority of *Neonectria* spp. lack sufficient genomic resources to resolve suspected cryptic species. Here, we report draft genomes and assemblies for *Neonectria magnoliae* NRRL 64651 and *Neonectria punicea* NRRL 64653.

## ANNOUNCEMENT

**Fig 1:**
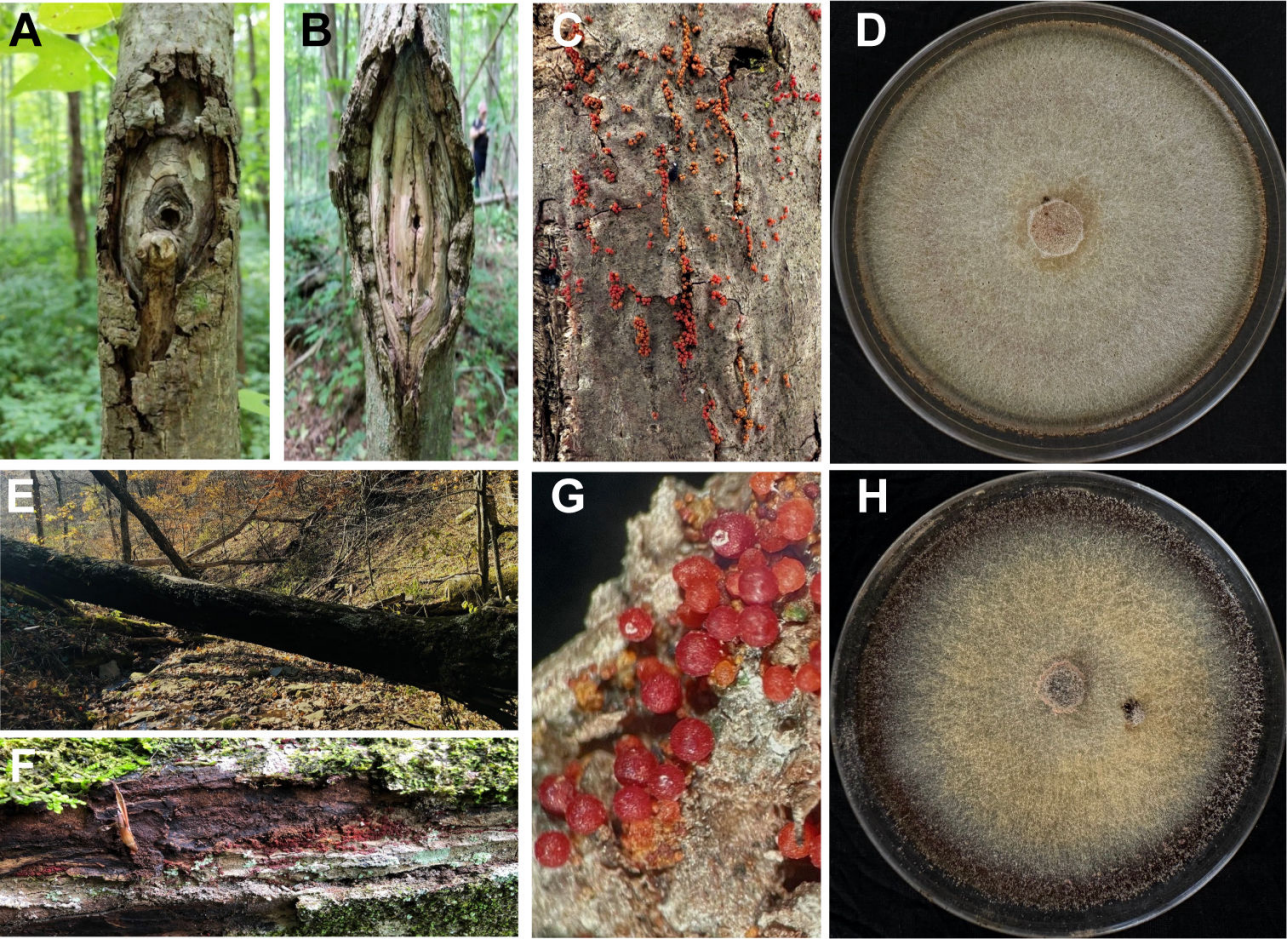
*In situ* and *in vitro* photos of *Neonectria magnoliae* (**A–D**) and *Neonectria punicea* (**E and F**) canker pathogens of woody angiosperms. *N magnoliae* produces both classic target-shaped or fusiform-shaped cankers (**A and B**) on healthy tulip-poplar (*Liriodendron tulipifera*) trees and inconspicuous cankers (**C**) on stressed, small-diameter understory trees. *N. punicea* produces inconspicuous cankers on dead and dying sugar maple (*Acer saccharum*) trees (**E and F**), identifiable primarily based on the occurrence of red perithecia (**C and G**). Single ascospore-derived cultures of *N. magnoliae* (**D**) and *N. punicea* (**H**) with characteristic *Cylindrocarpon*-morph colonies.

*Neonectria ditissima, N. faginata,* and *N. coccinea* cause lethal canker diseases of fruit (*Malus* and *Pyrus* spp.) and forest (*Fagus* spp.) trees and have been studied extensively (1–3). Since 2018, genomic data for these species and six additional *Neonectria* spp. have provided unique insights into their biology, pathogenicity, and host specialization [(4–10, Table 1)]. In 2020, *N. magnoliae,* a native canker pathogen of tulip-poplar (*Liriodendron tulipifera*) and Fraser magnolia (*Magnolia fraseri*), was formally described from isolates from West Virginia (Fig. 1; 11). During follow-up surveys in Pocahontas County, *N. punicea,* a species rarely documented in North America (12), was recovered from dead sugar maple (*Acer saccharum*; Fig. 1). Both *N. magnoliae* (*Nm*) and *N. punicea* (*Np*) co-occur with *N. ditissima* (*Nd*) and *N. faginata* (*Nf*) in eastern U.S. forests on three common hosts, *L. tulipifera* (*Nm,Nd*), *A. saccharum (Np,Nd),* and *F. grandifolia* (*Np,Nf*), yet their incidence and impact on these and other hosts are largely unexplored (1, 11–13). *Neonectria* and *Corinectria* canker diseases are of increasing concern (11, 14, 15), emphasizing an urgent need to fill critical data gaps; generating genomic resources for these species is fundamental to these efforts.

**TABLE 1:** Genome strain information and statistics for *Neonectria magnoliae* and *Neonectria punicea* with comparisons to other public genome resources

Species^*a*^	*N. magnoliae*	*N. punicea*	*N. punicea*	*N. faginata*	*N. coccinea*	*N. hederae*	*N. neomacrospora*	*N. ditissima*	*N. lugdunensis*	*N.* sp.	*C. fuckeliana*
Strain ID	NRRL 64651 /FERN 10531	NRRL 64653 /WVPC2	CBS 119724 /A.R. 3102	CBS 134246 / A.R. 4307	CBS 119158 /G.J.S. 98–114	CBS 714.97	KNNDK1	CBS 226.31 /IMI 113922	DSM 113088	DH2	CBS 125109 / G.J.S. 02–67
Year sampled / sequenced	2018/2024	2020/2024	1999/2018	2002/2021	1998/2021	1932/2018	2015/2021	1925/2021	1980/2024	2013/2018	2002/2021
Plant Host	*Liriodendron tulipifera*	*Acer saccharum*	*Frangula alnus*	*Fagus grandifolia*	*Fagus sylvatica*	*Hedera helix*	*Abies nordmanniana*	*Fagus sylvatica*	Unknown woody host	*Meconopsis grandis*	*Pinus radiata*
Location details	U.S.A.	U.S.A.	Austria	U.S.A.	Germany	Netherands	Denmark	Germany	Slovakia	China: Tibet	New Zealand
Coordinates (Lat., Long.)	39.034 N; −79.685 W	38.1913 N; −80.1963 W	-- ^*b*^	--	--	--	--	--	--	--	--
Sequencing Technology	Illumina NextSeq	Illumina NextSeq	Illumina MiSeq	Illumina MiSeq	Illumina MiSeq	Illumina MiSeq	PacBio	Illumina MiSeq	PacBio RSII	PacBio Sequel	Illumina MiSeq
Genome size (Mbp)	43.67	47.36	41.47	42.17	42.74	43.29	37.12	43.53	44.79	45.83	42.25
No. of scaffolds	408	1,077	1,779	522	571	508	12	1,274	15	43	737
Longest contig / scaffold (kbp)	1,157 / --	405 / --	-- / 342.6	-- / 709.7	-- / 532.9	-- / 802	-- / --	-- / 515.4	-- / --	5,080 / --	822.7
Scaffold N50 (kbp)	246.50	121.04	69.92	237.13	178.88	248.96	4,617.23	114.09	4,002.58	1,899.89	255.65
Scaffold L50	58	120	180	53	76	54	4	119	5	8	54
Avg coverage (x)	77	34	25	20	20	52	65	20	1	80	20
Total Illumina sequence (Gbp)^*c*^^,*d*^	3.7	1.8	--	0.0018	0.0053	--	--	0.0028	--	--	0.005
Total Illumina reads^*d*^	12,321,892	5,838,868	--	9,900,000	40,400,000	--	--	16,700,000	--	--	31,500,000
GC content (%)	52	52	53	53	52	50	53	52	52	53	50
Complete BUSCOs ascomycota_odb10 (%) / fungi_odb9 (%)^*d*^	1,696 (99.4)	1,694 (99.3)	-- / 1,412 (98.2)	-- / 1,353 (94.1)	-- / 1,424 (99.0)	-- / 1,429 (99.4)	--	-- / 1,415 (98.4)	--	(96.2) / --	-- / 1,428 (99.3)
Single-copy BUSCOs	1,686 (98.8)	1,686 (98.8)	--	--	--	--	--	--	--	--	--
Duplicated BUSCOs	10 (0.6)	9 (0.5)	--	--	--	--	--	--	--	--	--
Fragmented BUSCOs	3 (0.2)	2 (0.1)	--	--	--	--	--	--	--	--	--
Total genes (protein coding genes / tRNAs)^*d*^	12,569 (12,394/174)	13,388 (13,207/181)	13,180	12,991	12,941	11,966	11,291	13,669	---	13,606	11,446
GenBank assembly no.	GCA_037954305.1	GCA_037954315.1	GCA_003385315.1	GCA_019137275.1	GCA_019137265.1	GCA_003385265.1	GCA_917563905.1	GCA_019137815.1	GCA_041721585.1	GCA_003934905.1	GCA_019137255.1
WGS master record	JAZAVK000000000	JAZAVJ000000000	QGQA01000000	WPDD01000000	WPDF01000000	QGQB01000000	OU830638 -OU830649	WPDG01000000	JBGLZM010000000	RQWH01000000	WPDH01000000
SRA accession no.	SRR24938461	SRR24938484	--	SRR12873405	SRR12873403	--	--	SRR12873402	--	--	SRR12873401
BioSample accession no.	SAMN35642710	SAMN35646705	SAMN09242091	SAMN13483917	SAMN13483919	SAMN09242090	SAMEA9994693	SAMN13483920	SAMN43221455	SAMN10492166	SAMN13483921
Reference	This study	This study	4	6	6	4	7	6	N/A	5	6
Biosynthetic Gene Clusters (BGC)^*e*^											
T1PKS	12	10	9	8 (10)	10 (12)	7	12	11 (16)	12	14 (14)	14 (17)
T3PKS	1	1	1	1 (1)	1 (1)	1	1	1 (1)	1	1 (1)	1 (1)
NRPS	11	10	10	10 (18)	10 (20)	10	6	7 (23)	8	13 (13)	9 (22)
Terpenes	6	7	6	5 (5)	6 (7)	7	4	6 (6)	5	5 (5)	5 (5)
Other	5	5	6	7 (2)	6 (1)	7	9	9 (0)	7	11 (14)	4 (0)
Total	35	33	32	31 (33?)	33 (37?)	32	32	34 (39?)	33	44 (47)	33 (40?)

^*a*^ Seven additional total genomes are publicly available for *N. faginata* (two)*, N. coccinea* (one)*,* and *N. ditissima* (four), but are excluded from this table as only single representatives per species are provided for comparison.

^*b*^ “--”, Denotes missing data except for coordinates as they may exist but were not relevant to this study.

^*c*^ An Agilent 2100 Bioanalyzer system using an Agilent High-Sensitivity DNA Kit was used for sizing and quantitation of DNA sequencing libraries following the manufacturer’s protocols.

^*d*^ Denotes rows where data were extracted from the reference listed rather than from the NCBI Genome Assembly Accession page.

^*e*^ Antismash results: newly generated for this study (previously published results). Numbers followed by a “?” denote instances where total BCGs reported differ from the sum of the individual categories shown here. Results generated for this study used a “strict” detection strictness, whereas previously published studies did not include detection strictness in methods.

Single-ascospore cultures of *Nm* (NRRL 64651) and *Np* (NRRL 64653) were harvested from serial dilution platings of freshly collected individually macerated perithecia (Fig. 1). Cultures were grown out for ~2 weeks on potato dextrose agar at 20°C under ambient light conditions and genomic DNA extracted from harvested tissue with a Qiagen DNeasy PowerSoil Pro Kit using the manufacturer’s protocols. An Illumina NextSeq 1000 (Marshall University Genomics Core Facility, Huntington, WV), generated 12.3 M paired sequence reads or 3.7 Gb for *Nm* and 5.8 M paired sequence reads or 1.8 Gb for *Np*. The assembled genome for *Nm* strain NRRL 64651 was 43.67 Mbp (coverage, 77.6 x; *N*_50_, 246.50 kb; *L*_50_, 58; G + C content, 51.83%) and 47.36 Mbp (coverage, 34.2 x; *N*_50_, 121.04 kb; *L*_50_, 120; G + C content, 51.47%) for *Np* strain NRRL 64653 (Table 1). Both assemblies were cleaned of vector contamination and redundant contigs using SPAdes v3.15.2 running within AAFTF (v0.4.1) (16) with the steps trim, filter using fastp (17), and vectrim, sourpurge, and rmdup steps to remove contaminating contigs in the assembly. Assemblies were further corrected by five rounds of polishing with Pilon (v1.24) with the Illumina reads. Genome annotation was performed with funannotate (v1.8.15; (18)) utilizing alignment of proteins in UniProt and BUSCO with sordariomycetes_odb10 for training. tRNA genes were predicted using tRNAscan-SE v2.0.9 (19). BUSCO v5.4.4 (20), using the ascomycota_odb10 data set (21), identified 1,696 complete markers (99.4%) in *Nm* and 1,694 (99.3%) in *Np* (Table 1). Default parameters were used or when specified, available in the pipeline code, parameters, and logfiles at GitHub and Zenodo (22). The final genome annotations included a total of 12,394 protein-coding genes (PCGs) and 174 tRNAs for *Nm* and 13,207 PCGs and 181 tRNAs for *Np* (4–9; Table 1). AntiSMASH (v5.0; (23)) predicted 32 and 35 biosynthetic gene clusters (BGCs) for *Nm* and *Np*, respectively. Genome size, gene counts, and BGCs agreed with published statistics for other *Neonectria* spp. (Table 1).

## Data Availability

This whole-genome shotgun project has been deposited at DDBJ/ENA/GenBank under the accession numbers JAZAVK010000000 and JAZAVJ010000000. Sequence reads were deposited under SRA project accession numbers SRR24938461 and SRR24938484 and BioProject accession numbers PRJNA980700 and PRJNA980721.
